# Antiallodynic effects of alpha lipoic acid in an optimized RR-EAE mouse model of MS-neuropathic pain are accompanied by attenuation of upregulated BDNF-TrkB-ERK signaling in the dorsal horn of the spinal cord

**DOI:** 10.1002/prp2.137

**Published:** 2015-05-04

**Authors:** Nemat Khan, Richard Gordon, Trent M Woodruff, Maree T Smith

**Affiliations:** 1 Center for Integrated Preclinical Drug Development, University of QueenslandSt Lucia Campus, Brisbane, Queensland, 4072, Australia; 2 School of Pharmacy, University of Queensland, Pharmacy Australia Center of ExcellenceWoolloongabba, Brisbane, Queensland, 4102, Australia; 3 The School of Biomedical Sciences, University of QueenslandSt Lucia Campus, Brisbane, Queensland, 4072, Australia

**Keywords:** Alpha lipoic acid, analgesia, brain-derived neurotrophic factor, mechanical allodynia, multiple sclerosis, neuropathic pain, relapsing-remitting experimental autoimmune encephalomyelitis, tyrosine kinase B

## Abstract

Neuropathic pain may affect patients with multiple sclerosis (MS) even in early disease. In an experimental autoimmune encephalomyelitis (EAE)-mouse model of MS, chronic alpha lipoic acid (ALA) treatment reduced clinical disease severity, but MS-neuropathic pain was not assessed. Hence, we investigated the pain-relieving efficacy and mode of action of ALA using our optimized relapsing-remitting (RR)-EAE mouse model of MS-associated neuropathic pain. C57BL/6 mice were immunized with MOG_35-55_ and adjuvants (Quil A and pertussis toxin) to induce RR-EAE; sham-mice received adjuvants only. RR-EAE mice received subcutaneous ALA (3 or 10 mg kg^−1^ day^−1^) or vehicle for 21 days (15–35 d.p.i.; [days postimmunization]); sham-mice received vehicle. Hindpaw hypersensitivity was assessed blinded using von Frey filaments. Following euthanasia (day 35 d.p.i.), lumbar spinal cords were removed for immunohistochemical and molecular biological assessments. Fully developed mechanical allodynia in the bilateral hindpaws of vehicle-treated RR-EAE mice was accompanied by marked CD3^+^ T-cell infiltration, microglia activation, and increased brain-derived neurotrophic factor (BDNF)-tyrosine kinase B (TrkB) signaling in the dorsal horn of the lumbar spinal cord. Consequently, phospho-ERK, a marker of central sensitization in neuropathic pain, was upregulated in the spinal dorsal horn. Importantly, hindpaw hypersensitivity was completely attenuated in RR-EAE mice administered ALA at 10 mg kg^−1^ day^−1^ but not 3 mg kg^−1^ day^−1^. The antiallodynic effect of ALA (10 mg kg^−1^ day^−1^) was associated with a marked reduction in the aforementioned spinal dorsal horn markers to match their respective levels in the vehicle-treated sham-mice. Our findings suggest that ALA at 10 mg kg^−1^ day^−1^ produced its antiallodynic effects in RR-EAE mice by reducing augmented CD3^+^ T-cell infiltration and BDNF-TrkB-ERK signaling in the spinal dorsal horn.

## Introduction

Multiple sclerosis (MS) is a chronic inflammatory-demyelinating disease of the central nervous system (CNS). Apart from motor impairment, MS is often associated with sensory changes (Compston and Coles 2008). Central neuropathic pain (CNP), that may affect patients with MS even early in the disease course, is mostly persistent in nature and unaffected by the clinical disease course (Kalia and O’Connor 2005; Osterberg et al. 2005). Similarly, the time course for development of CNP behaviors in experimental autoimmune encephalomyelitis (EAE)-rodent models is discordant with that for development of EAE-induced motor impairments (Khan and Smith 2014). EAE-induced motor deficits are predominantly correlated with pathobiologic changes in the ventral horn of the spinal cord and/or dorsal funicular white matter (Wu et al. 2008). By contrast, neuropathic pain behaviors in rodent models of CNP are associated with pathobiologic mechanisms in sensory neurons in the dorsal horn (laminae I–II) of the spinal cord (Khan et al. 2014; Watson et al. 2014). Recommended drug treatments for the relief of peripheral neuropathic pain conditions (Dworkin et al. 2010) are often ineffective for alleviating MS-associated CNP (Khan and Smith 2014). Hence, new and well-tolerated analgesic treatments for improved relief of this condition, are an unmet medical need.

Alpha lipoic acid (ALA), a nutraceutical compound known for antioxidant properties has been shown recently to produce significant alleviation of peripheral neuropathic pain in rodent models of painful diabetic neuropathy (Morgado et al. 2011) and cancer chemotherapy-induced peripheral neuropathy (CIPN), when administered by chronic and acute dosing regimens, respectively (Trevisan et al. 2013). In other work using EAE rodent models, chronically administered ALA attenuated clinical disease severity (Morini et al. 2004; Wang et al. 2013), but its effect on MS-associated CNP was not investigated.

Hence, the present work was designed to assess the potential pain-relieving efficacy and mode of action of ALA administered chronically according to an intervention protocol, using an optimized relapsing-remitting experimental autoimmune encephalomyelitis (RR-EAE) mouse model of MS-induced neuropathic pain developed by our group (Khan et al. 2014). Notably, our findings are novel showing that once-daily subcutaneous (s.c.) administration of ALA at 3 or 10 mg kg^−1^ to RR-EAE mice for 21 consecutive days, evoked dose-dependent alleviation of mechanical hypersensitivity in the bilateral hindpaws. The pain-relieving mode of action involved a significant reduction in augmented CD3^+^ T-cell infiltration and microglial activation in the dorsal horn of the lumbar (L4-L6) spinal cord. This in turn reduced EAE-induced upregulation of expression levels of BDNF, tyrosine kinase B (TrkB), and phosphorylated-p44/p42 mitogen-activated protein kinase (pp44/pp42 MAPK; also called pERK), a marker of BDNF/TrkB downstream signaling, in the lumbar spinal dorsal horn of RR-EAE mice to match the respective levels in vehicle-treated sham-control mice. To the best of our knowledge, this is the first study to evaluate the potential antiallodynic effects of ALA in a rodent model of MS-induced neuropathic pain, and we show that pain-relief appears to be mediated by attenuation of upregulated BDNF-TrkB-ERK signaling in the spinal dorsal horn of RR-EAE mice.

## Materials and Methods

### Animals

Female C57BL/6 mice aged 4–6 weeks were from the University of Queensland Biological Resources. Mice were housed in groups of 6–8 per cage in a temperature-controlled facility (22–23°C) with a 12/12 h light/dark cycle. Rodent chow and water were available ad libitum. Ethics approval was from the Animal Ethics Committee of the University of Queensland. Experiments complied with the requirements of the Australian Code of Practice for the Care and Use of Animals for Scientific Purposes (NHMRC (National Health and Medical Research Council) 2004, 2013; ) and the ARRIVE guidelines (Kilkenny et al. 2010).

### EAE induction and clinical disease scoring

Our optimized RR-EAE mouse model of MS-associated neuropathic pain is novel and it is devoid of confounding motor deficits in the hindpaws (Khan et al. 2014). Briefly, mice were immunized with 200 *μ*g of MOG_35-55_ (Mimotopes, Clayton, Victoria, Australia) mixed with a solution of Quil A (45 *μ*g) in 100 *μ*L of phosphate-buffered saline (PBS) (Sigma-Aldrich, Sydney, New South Wales, Australia). Mice then received an intraperitoneal (i.p.) injection of pertussis toxin at 250 ng (Sigma-Aldrich) in PBS (1 ng *μ*L^−1^) and this was repeated 48 h later (Khan et al. 2014). Sham-mice (control group) received adjuvants only (Quil A and pertussis toxin). Clinical disease scoring of RR-EAE and sham-mice was undertaken once-daily in a blinded manner using a 5-point scale with half-point gradations (Table1) (Khan et al. 2014; Peiris et al. 2007). RR-EAE clinical disease was classified as present by clinical scores ≥1, whereas clinical scores ≤0.5 were regarded as disease remission or absence. General health and body weights of all mice were assessed prior to immunization and once-daily thereafter in a blinded manner until the completion of study.

**Table 1:** EAE clinical disease scoring paradigm.

Score	Description
0	Normal behavior
0.5	Limpness of the distal tail region and hunched appearance
1	Completely limp tail or developing weakness in the hindlimbs
1.5	Limp tail and distinct hindlimbs weakness recognized by unsteady gait and poor grip of hindlimbs during hanging on cage underside
2	Limp tail with unilateral partial hindlimb paralysis
2.5	Limp tail and partial paralysis of bilateral hindlimbs
3	Complete paralysis of bilateral hindlimbs
3.5	Complete bilateral hindlimbs paralysis and unilateral forelimb paralysis
4	Quadriplegia

EAE, experimental autoimmune encephalomyelitis.

### Test compound dosing regimen

ALA was from GeroNova Research Inc. (Richmond, CA) and supplied as the water-soluble sodium salt of the R-enantiomer of ALA. Dosing solutions of ALA were prepared in sterile water for injection (Pfizer, West Ryde, New South Wales, Australia).

**Figure 1:**
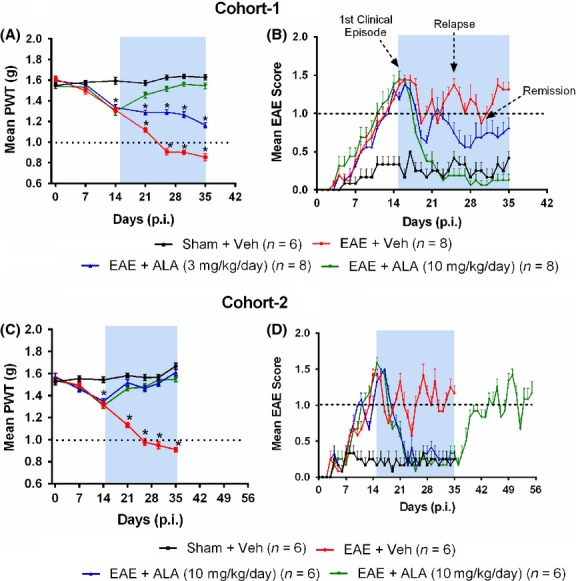
Hindpaw hypersensitivity and clinical disease in our optimized RR-EAE mouse model of MS-induced neuropathic pain were progressively alleviated by once-daily administration of single s.c. bolus doses of ALA administered according to an intervention protocol at 15–35 d.p.i. (A, C) For vehicle-treated RR-EAE mice (Cohorts 1 and 2), mean (±SEM) hindpaw PWTs were significantly lower than those for vehicle-treated sham-mice (*F*_(3,6,18/336_ = 142.2, 73.43, 43.66; Cohort-1 and *F*_(3,6,18/264_ = 239.1, 25.11, 34.32; Cohort-2; *P *<* *0.05). Chronic ALA treatment at 3 or 10 mg kg^−1^ day^−1^ (15–35 d.p.i.) evoked dose-dependent attenuation of hindpaw hypersensitivity in RR-EAE mice, whereas for vehicle-treated RR-EAE mice, mechanical allodynia was fully developed (PWTs ≤ 1 g) in the bilateral hindpaws from 26 d.p.i. onward (*P *<* *0.05). After cessation of ALA-treatment (10 mg kg^−1^ day^−1^) at 35 d.p.i. in Cohort-2 RR-EAE mice, there was temporal development of mechanical allodynia in the bilateral hindpaws that was fully developed at study completion (55 d.p.i.). *P *<* *0.05 (two way ANOVA, post hoc: Bonferroni). (B) and (D) For vehicle-treated RR-EAE mice (Cohorts 1 and 2), clinical disease onset was at 10–13 d.p.i. and it exhibited a relapsing-remitting profile (*F*_(4,144_ = 34.89; Cohort-1 and *F*_(4,164_ = 33.05; Cohort-2; *P *<* *0.05). By contrast, vehicle-treated sham-mice did not exhibit EAE-clinical disease (mean ± SEM clinical score >1 a.u.). For both cohorts of RR-EAE mice, ALA at 10 mg kg^−1^ day^−1^ but not 3 mg kg^−1^ day^−1^ successfully prevented clinical disease relapse. After cessation of ALA-treatment (10 mg kg^−1^ day^−1^) at 35 d.p.i. in Cohort-2 RR-EAE mice, clinical disease recurred. The shaded areas on figure panels highlight the chronic ALA or vehicle treatment period (15–35 d.p.i.). *P <* *0.05 (Kruskal–Wallis multiple comparison, post hoc: Dunn’s test). ALA, alpha lipoic acid; EAE, experimental autoimmune encephalomyelitis; PWT, Paw withdrawal threshold; p.i., postimmunization; Veh, vehicle. MS, multiple sclerosis.

RR-EAE mice received once-daily s.c. injections of ALA at 3 or 10 mg kg^−1^ day^−1^, or vehicle for 21 consecutive days commencing at 15 d.p.i., as the first episode of clinical disease had occurred in all EAE mice by day 15. Sham-control mice received once-daily s.c. vehicle injections for the same treatment period. Solutions of ALA or vehicle were prepared freshly each day and masked before administration to animals in a blinded manner. All drug treatments were administered between 09.00 am and 11.00 am. The total number of sham- and RR-EAE mice (in Cohorts 1 and 2) treated with vehicle or ALA is shown in Figure1. To maintain blinding, equal numbers of animals were included in each group. Animals were randomized in a blinded manner and received masked solutions of ALA or vehicle by the first person.

The drug and molecular target nomenclature used herein is in accordance with that recommended by Alexander et al. (2013).

### Assessment of antiallodynic effects evoked by chronic ALA in RR-EAE mice

Calibrated von Frey filaments (Stoelting Co., Wood Vale, IL) were used according to the up–down method (Chaplan et al. 1994) to assess paw withdrawal thresholds (PWTs) in the bilateral hindpaws of RR-EAE mice relative to sham-mice by a blinded tester. Briefly, baseline PWTs were measured prior to immunization on day 0 and once-weekly thereafter to define the time course for temporal development of mechanical allodynia. For assessment of the antiallodynic effects of ALA (or vehicle) in RR-EAE mice, hindpaw PWTs were measured once-weekly at 0.5–0.75 h post-dosing. This was the time of peak effect observed in pilot experiments and it is in agreement with the time of peak antinociception produced by single bolus doses of ALA in a rat model of CIPN (Trevisan et al. 2013). Hindpaw PWTs for sham-mice were assessed concurrently with the corresponding cohort of RR-EAE mice by a blinded tester.

### Chronic ALA treatment: EAE clinical disease profiling

The effect of ALA on EAE clinical disease progression was assessed by measuring clinical disease scores once-daily at 0.5–0.75 h post-dosing. Clinical disease scores for sham-mice administered the chronic vehicle dosing regimen were also assessed concurrently with the corresponding cohort of RR-EAE mice in a blinded manner.

### Ex-vivo analysis: tissue collection and preparation

RR-EAE mice administered the chronic ALA (10 mg kg^−1^ day^−1^) or vehicle treatment, as well as sham-mice (administered the chronic vehicle dosing regimen *n* = 4/group for each technique), were euthanized with an overdose (∼300 mg kg^−1^; i.e. 1 mL/kg of 325 mg/mL, i.p.) of pentobarbitone (Lethabarb ®; Virbac, Milperra, New South Wales, Australia) at 0.5–0.75 h post-dosing on the last treatment day (35 d.p.i.). Lumbar (L4-L6) spinal cord tissues were removed for ex-vivo mode of action analyses using immunohistochemistry (IHC), western blotting, and quantitative real-time PCR (RTqPCR). For IHC, mice were perfused with 4% paraformaldehyde (Sigma-Aldrich) in ice-cold 1 × PBS (pH 7.4; ∼100 mL/mouse) prior to removal of lumbar spinal cord tissues. Transverse cryosections (10–12 *μ*m) were prepared and mounted on Superfrost Plus® slides (Thermo Fisher Scientific, Scoresby, Victoria, Australia) prior to immunostaining, as per our previous report (Khan et al. 2014). For western blot and RTqPCR, lumbar (L4–L6) spinal cord tissues were collected without perfusion and immediately stored at −80°C prior to further processing as described below.

### Immunohistochemistry

Sections of lumbar spinal cord were permeabilized with ice-cold acetone and incubated in blocking buffer containing 10% normal goat serum (Invitrogen*,* Mulgrave, Victoria, Australia), 0.2% Triton-X (Sigma-Aldrich), and 0.05% Tween-20 in PBS solution prior to immunostaining (Khan et al. 2014). Briefly, sections were incubated overnight with the relevant primary antibody at ∼4–8°C and on the following day, were incubated with the corresponding secondary antibody for ∼1 h at room temperature. Later, sections were stained with 4′,6-diamidino-2-phenylindole dihydrochloride (DAPI; Invitrogen) for 5 min and mounted with ProLong® Gold antifade reagent (Invitrogen) prior to imaging. Following staining, sections were washed with PBST (2 × 10 min). Primary antibodies used were: monoclonal rat anti-CD3 (1:200; Serotec, Kidlington, UK); monoclonal rat anti-mouse CD11b (1:200; Serotec); polyclonal rabbit anti-BDNF (1:200; Millipore, Kilsyth, Victoria, Australia); monoclonal rat anti-TrkB (1:50; R&D Systems, Minneapolis, MN) and monoclonal rabbit anti-pp44/pp42 MAPK (Cell Signaling Technology, Inc., Danvers, MA). Additional antibodies used for colocalization experiments were: monoclonal rabbit anti-CD3 (1:200; Abcam, Cambridge, MA); polyclonal rabbit anti-Iba-1 (1:600, Wako, Osaka, Japan) and monoclonal mouse anti-NeuN Alexa Flour 488 conjugated (1:50, Millipore). Secondary antibodies used included: Cy3-goat anti-rat (1:800; Jackson ImmunoResearch, Westgrove*,* PA); Cy3-goat anti-rabbit (1:1000; Jackson); Alexa Flour 488 goat anti-rat (1:500; Invitrogen) or Alexa Flour 488 goat anti-rabbit (1:1000; Invitrogen). Primary and secondary antibodies were diluted in PBST containing 2% normal goat serum. Omission of primary antibodies on negative control sections during each staining procedure resulted in a complete absence of immunofluoresence (IF).

### Western blotting

Lumbar spinal cord tissues were homogenized individually for each animal and protein extracted using radio-immunoprecipitation assay buffer. From each extract, 40 *μ*g of total protein was loaded onto 4–20% gradient precast polyacrylamide gels (Bio-Rad, Gladesville, New South Wales, Australia). Each gel was transferred to a 0.2 *μ*m nitrocellulose membrane (Bio-Rad) and blocked in Odyssey™ blocking buffer (LI-COR, Lincoln, NE). For phospho-specific antibodies, blocking buffer comprised 5% bovine serum albumin/0.1% Tween-20. Membranes were incubated overnight at ∼4°C with primary antibodies against BDNF (1:500), TrkB (1:300), Iba-1 (1:1000), pp44/pp42 MAPK (pERK) (1:1000) or p44/p42 MAPK (total ERK) (1:1000). Primary antibodies were the same as listed in the preceding section except for BDNF (N-20) from Santa Cruz Biotechnology (Dallas, Texas) that has been validated to identify all three isoforms of BDNF (Tongiorgi et al. 2012). Monoclonal mouse anti-GAPDH (1:8000; Novus, Littleton, CO) was used as loading control. Membranes were washed using PBST (3 × 5 min) and incubated in appropriate infrared dye-conjugated secondary antibodies (LI-COR) (1:8000) for ∼1 h at room temperature. Membranes were visualized using an Odyssey infrared scanner (LI-COR).

### Quantitative real-time PCR

Absolutely RNA Miniprep Kits (Agilent, Santa Clara, CA) were used to isolate total RNA individually from lumbar spinal cord tissue from each mouse. Complementary DNA (cDNA) was reverse-transcribed from total RNA using high-capacity cDNA reverse transcription kits (Applied Biosystems, Mulgrave, Victoria, Australia). Quantitative BDNF and TrkB messenger RNA (mRNA) expression levels were analyzed using SYBR® Green PCR master mix (Applied Biosystems) and specific primers. The specificity of the real-time PCR reaction was confirmed using melting curve analysis. Primers used were (1) 18S-forward: CCCTCCAATGGATCCTCGTT; 18S-reverse: TCGAGGCCCTGTAATTGGAA (2) BDNF QuantiTect® primer assay, Mm_Bdnf_1_SG (Qiagen, Pty Ltd., Victoria, Australia) and (3) TrkB QuantiTect® primer assay, Mm_Ntrk2_vb.1_SG (Qiagen) containing a mixture of forward and reverse primers.

### Mouse plasma biochemistry and organ weights

Please see supplementary methods for method descriptions.

### Data analyses

#### Behavioral data

Graphical data are presented as mean ± standard error of the mean (±SEM). Clinical scores were the mean (±SEM) score for each group of mice at each time point. Hindpaw PWT values are the mean (±SEM) of three measurements taken at least 5 min apart for both hindpaws combined.

#### Immunohistochemistry data

IIHC images of at least 3–4 lumbar spinal dorsal horn sections (>100 *μ*m apart) per animal from each treatment group (*n* = 3–4/group) were used for quantitative analysis or colocalization studies in vehicle-treated RR-EAE mice as appropriate. Laminae I–II of the spinal dorsal horn were targeted for all IHC analyses (Fig. S2). Densitometric counts were quantified using Axiovision Rel. v4.8 software (Carl Zeiss, Göttingen, Germany) in a blinded manner (Khan et al. 2014). Data are expressed as fold-changes in fluorescence intensity for sections from RR-EAE mice treated with ALA (10 mg kg^−1^ day^−1^) or vehicle, relative to the corresponding data for vehicle-treated sham-mice.

#### Western blot data

Image Studio Lite v4.0 software (LI-COR) was used to quantify immunoreactive band intensities in western blots for each treatment group (*n* = 3/group). For each protein of interest, band intensity was normalized relative to GAPDH (Jain et al. 2012) to give relative expression levels and fold-changes between ALA- and vehicle-treated RR-EAE mice compared with the vehicle-treated sham-group.

#### RTqPCR data

For RTqPCR, mRNA expression levels were normalized relative to 18S, for each treatment group (*n* = 3–4/group). For the ALA- and vehicle-treated RR-EAE mice, mRNA levels were quantified relative to that for vehicle-treated sham-mice using the ΔΔC_T_ method (Schmittgen and Livak 2008). The fold-changes for each target of interest were determined using the 2^−ΔΔCT^ method (Schmittgen and Livak 2008).

#### Mouse plasma biochemistry and organ weights

Mouse plasma biochemistry parameters are reported as mean (±SEM). Organ weights (kidneys and liver) are presented as relative to respective body weights on the day of euthanasia (Lee et al. 2014) (Table2).

**Table 2:** Mean (±SEM) plasma biochemical parameters and organ weights in mice.

	Sham + Veh	EAE + Veh	EAE + ALA (10 mg kg^−1^ day^−1^)
Biochemical parameters (mean ± SEM)			
ALT (U/L)	29.25 ± 4.6	24.75 ± 9.1	27.5 ± 5.9
AST (U/L)	394.8 ± 53.3	324.5 ± 73.3	313.0 ± 85.7
ALP (U/L)	31.0 ± 10.9	38.8 ± 9.9	38.8 ± 3.3
Total bilirubin, *μ*mol/L	2.5 ± 0.5	6.3 ± 2.5	2.0 ± 0.0
Urea mmol/L	4.4 ± 0.2	3.7 ± 0.5	4.6 ± 0.1
Creatinine*, *μ*mol/L	BLOQ	BLOQ	BLOQ
Organ weights (mg; mean ± SEM)			
Body weights (at euthanasia)	20.2 ± 0.4	19.6 ± 0.3	19.5 ± 0.3
Liver			
Absolute	947 ± 40.0	925 ± 35.9	894.5 ± 37.9
Relative	4.68 ± 0.14	4.71 ± 0.15	4.57 ± 0.19
Kidneys			
Absolute	247.6 ± 10.1	262.1 ± 8.1	260.1 ± 10.0
Relative	1.22 ± 0.03	1.33 ± 0.02	1.33 ± 0.05

ALA, alpha lipoic acid; ALT, alanine transaminase; AST, aspartate transaminase; ALP, alkaline phosphatise; EAE, experimental autoimmune encephalomyelitis; SEM, standard error mean; Veh, vehicle.

* Below lower limit of quantification (BLOQ) which was <20 *μ*mol/L; the normal range for C57BL6 mice is ≤20 *μ*mol/L (Zhou and Hansson 2004).

### Statistical analyses

Statistical analyses were performed using GraphPad Prism™ v6.04 (GraphPad Software, La Jolla, USA). Repeated measures two-way analysis of variance (ANOVA) followed by the Bonferroni test was used to analyze pain behavioral end points. The Kruskal–Wallis test with the multiple comparison post hoc Dunn’s test, was used to analyze clinical scores for the various groups of mice, consistent with previous work by others (Fleming et al. 2005; Amor and Baker 2012). The one-way ANOVA followed by Tukey’s multiple comparison test, was used to compare intergroups differences for IHC, western blot and RTqPCR data, plasma biochemistry data, and organ weights. The statistical significance criterion was *P *<* *0.05.

## Results

### RR-EAE mice: mechanical hypersensitivity in the bilateral hindpaws

Mean (±SEM) hindpaw PWTs for vehicle-treated RR-EAE mice of Cohorts 1 and 2 were significantly decreased *c.f*. PWTs for vehicle-treated sham-mice from 14 d.p.i. until study completion at 35 d.p.i. (*F*_(3,6,18/336_ = 142.2, 73.43, 43.66; Cohort-1 and *F*_(3,6,18/264_ = 239.1, 25.11, 34.32; Cohort-2; *P *<* *0.05). Additionally, mechanical allodynia was fully developed (PWTs ≤ 1 g) in the bilateral hindpaws of vehicle-treated RR-EAE mice from 26 d.p.i. (*P *<* *0.05) which persisted until study completion at 35 d.p.i. (Fig.1).

### Chronic ALA treatment alleviates hindpaw hypersensitivity in RR-EAE mice

In RR-EAE mice, once-daily s.c. administration of ALA at 3 or 10 mg kg^−1^ day^−1^ for 21 consecutive days (15–35 d.p.i.) evoked dose-dependent alleviation of mechanical hypersensitivity in the bilateral hindpaws. Specifically, mean (±SEM) hindpaw PWTs for RR-EAE mice (Cohorts 1 and 2) administered the chronic dosing regimen of ALA at 10 mg kg^−1^ day^−1^, did not differ significantly (*P *>* *0.05) from the corresponding groups of vehicle-treated sham-mice at 35 d.p.i. However, by 3 weeks after cessation of ALA at 10 mg kg^−1^ day^−1^ in Cohort-2 RR-EAE mice at 55 d.p.i., mechanical allodynia was again fully developed in the bilateral hindpaws of these mice (Fig.1).

### RR-EAE disease course

For RR-EAE mice from Cohorts 1 and 2, disease onset (clinical scores ≥1 arbitrary units [a.u.]) was between 8 and 14 d.p.i. This was followed by mild relapsing-remitting disease with mean (±SEM) peak clinical scores of 1.4 (±0.06) a.u. during the chronic vehicle treatment regimen (15–35 d.p.i.). Importantly, clinical disease progression in vehicle-treated RR-EAE mice differed significantly from the corresponding cohorts of vehicle-treated sham-mice (*F*_(4,144_ = 34.89; Cohort-1 and *F*_(4,164_ = 33.05; Cohort-2; *P *<* *0.05) that did not develop clinical disease (mean ± SEM ≤ 0.5 a.u.) over the study period (Fig.1), mirroring our previous work (Khan et al. 2014).

### Chronic ALA treatment arrests clinical disease relapse in RR-EAE mice

RR-EAE mice (Cohorts 1 and 2) treated with ALA at 3 or 10 mg kg^−1^ day^−1^ for 21 consecutive days commencing on day 15 d.p.i did not exhibit clinical disease relapses. Specifically, the clinical disease patterns in Cohorts 1 and 2 of RR-EAE mice treated with ALA at 10 mg kg^−1^ day^−1^ were comparable (*P *>* *0.05) to those from the corresponding vehicle-treated sham-mice at the end of treatment (35 d.p.i.) (Fig.1). However, by 9 days after cessation of ALA treatment at 10 mg kg^−1^ day^−1^ in Cohort 2 RR-EAE mice (44 d.p.i.), there was a recurrence of relapsing-remitting clinical disease that persisted until study completion at 55 d.p.i. (Fig.1).

### Chronic ALA treatment of RR-EAE mice inhibits putative markers of MS-induced neuropathic pain

#### CD3^+^ T-cell infiltration

**Figure 2:**
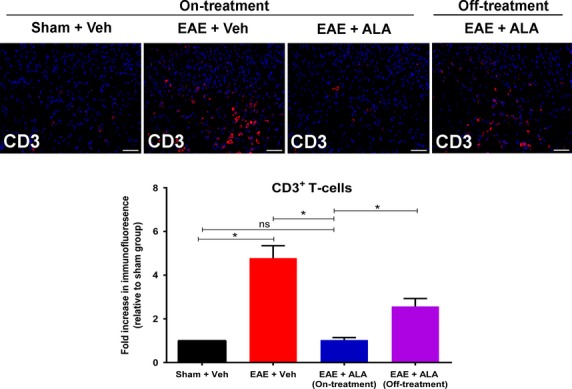
Immunohistochemical analysis of the extent of CD3^+^ T-cell infiltration into the dorsal horn of lumbar (L4-L6) spinal cord sections from RR-EAE mice administered ALA at 10 mg kg^−1^ day^−1^ or vehicle, for 3-weeks (15–35 d.p.i.) relative to sham-mice administered vehicle by the same dosing schedule. CD3^+^ T-cell immunofluorescence (IF) in lumbar spinal dorsal horn was ∼4.7-fold higher (*F*_(3,44)_ = 26.44; *P *<* *0.05) for vehicle-treated RR-EAE mice *c.f*. that for vehicle-treated sham-mice. By contrast, CD3^+^ T-cell IF in lumbar spinal dorsal horn of RR-EAE mice administered ALA at 10 mg kg^−1^ day^−1^ for 21 consecutive days did not differ significantly (*F*_(3,44)_ = 26.44; *P *>* *0.05) from the corresponding data for vehicle-treated sham-mice. After cessation of ALA-treatment (10 mg kg^−1^ day^−1^) at 35 d.p.i. in RR-EAE mice, CD3^+^ T-cell IF in lumbar spinal dorsal horn at day 55  d.p.i. was again increased by ∼2.5-fold (*F*_(3,44)_ = 26.44; *P *<* *0.05) *P <* *0.05 ((one-way ANOVA followed by Tukey’s multiple comparison test) to match the corresponding levels for vehicle-treated RR-EAE mice). Scale bars are 50 *μ*m. EAE, experimental autoimmune encephalomyelitis, Veh, vehicle; ALA, alpha lipoic acid.

As the role of reactive T-cells in MS-associated neuropathic pain is unknown (Khan and Smith 2014), we examined CD3^+^ T-cell IF in the lumbar spinal dorsal horn of RR-EAE mice. Specifically, CD3^+^ T-cell IF was ∼4.7-fold higher (*F*_(3,44)_ = 26.44; *P *<* *0.05) in vehicle-treated RR-EAE mice exhibiting neuropathic pain behavior at 35 d.p.i. *c.f*. the respective levels in vehicle-treated sham-mice. For RR-EAE mice administered ALA at 10 mg kg^−1^ day^−1^ for 21-days, CD3^+^ T-cell IF in the lumbar spinal dorsal horn did not differ significantly (*P *>* *0.05) from that for vehicle-treated sham-mice. However, by 3 weeks after cessation of ALA treatment at 10 mg kg^−1^ day^−1^ in Cohort-2 RR-EAE mice at 55 d.p.i., CD3^+^ T-cell IF in the spinal dorsal horn was again increased to ∼2.5-fold higher (*F*_(3,44)_ = 26.44; *P *<* *0.05) than in vehicle-treated sham-mice (Fig.2).

#### Microglial activation

**Figure 3:**
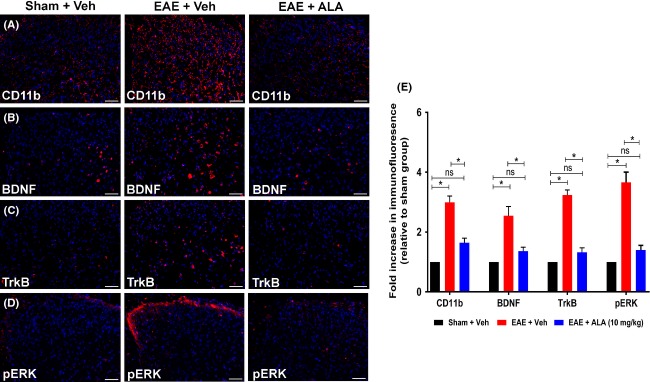
Immunohistochemical (IHC) analysis of CD11b, BDNF, TrkB, and pERK in the dorsal horn of lumbar (L4-L6) spinal cord sections from RR-EAE mice administered ALA at 10 mg kg^−1^ day^−1^ or vehicle, for 3 weeks relative to the corresponding data for vehicle-treated sham-mice. For vehicle-treated RR-EAE mice, there was a significant increase in the (A, E) extent of microglia/macrophage (CD11b) activation (approximately threefold; *F*_(2,33)_ = 45.57; *P *<* *0.05), as well as expression levels of (B, E) BDNF (∼2.5-fold; *F*_(2,33)_ = 15.84; *P *<* *0.05); (C, E) TrkB (∼3.2-fold; *F*_(2,33)_ = 71.52; *P *<* *0.05) and (D, E) pERK (∼3.6-fold; *F*_(2,33)_ = 42.49; *P *<* *0.05) *c.f*. the respective data for the lumbar spinal cord of vehicle-treated sham-mice. For RR-EAE mice treated with ALA (10 mg kg^−1^ day^−1^), lumbar spinal cord expression levels of CD11b, BDNF, TrkB, and pERK did not differ significantly (*P *>* *0.05) from the respective data for lumbar spinal cord of vehicle-treated sham mice, *P <* *0.05 (one-way ANOVA followed by Tukey’s multiple comparison test). Scale bars represent 50 *μ*m. EAE, experimental autoimmune encephalomyelitis, Veh, vehicle; ALA, alpha lipoic acid; BDNF, brain-derived neurotrophic factor; TrkB, tyrosine kinase B; ANOVA, analysis of variance.

**Figure 4:**
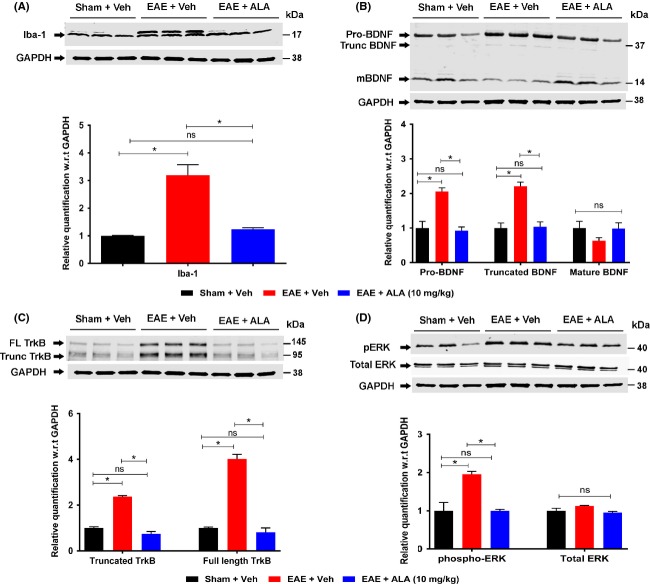
Western blot analyses of microglial activation (Iba-1), as well as expression levels of BDNF, TrkB, pERK, and total ERK in lumbar (L4-L6) spinal cord of RR-EAE mice administered ALA at 10 mg kg^−1^ day^−1^ or vehicle, for 3 weeks relative to the respective data for the lumbar spinal cord of vehicle-treated sham-mice. Specifically, for vehicle-treated RR-EAE mice, there was a significant increase in (A) the extent of microglial (Iba-1) activation (approximately threefold; *F*_(2,6)_ = 29.82; *P *<* *0.05) (B) expression levels of the BDNF isoforms, Pro-BDNF (approximately twofold; *F*_(2,6)_ = 20.31; *P *<* *0.05) and truncated BDNF (∼2.2-fold; *F*_(2,6)_ = 24.90; *P *<* *0.05) but not mature BDNF (*F*_(2,6)_ = 1.79; *P *>* *0.05) (C) expression levels of the TrkB isoforms, truncated TrkB (∼2.3-fold; *F*_(2,6)_ = 164.7; *P *<* *0.05) and full-length TrkB (approximately fourfold; *F*_(2,6)_ = 125.2; *P *<* *0.05), as well as (D) expression levels of phospho-ERK (*F*_(2,6)_ = 16.43; *P *<* *0.05) but not total ERK (*F*_(2,6)_ = 4.066; *P *>* *0.05) *c.f*. the respective data for lumbar spinal cord of vehicle-treated sham-mice. For RR-EAE mice treated with ALA (10 mg kg^−1^ day^−1^) for 21 days, there were no significant differences (*P *>* *0.05) in expression levels of any of the aforementioned lumbar spinal cord markers from the respective data for the lumbar spinal cord of vehicle-treated sham-mice *P <* *0.05 (one-way ANOVA followed by Tukey’s multiple comparison test). EAE, experimental autoimmune encephalomyelitis; FL, full-length; mBDNF, mature-BDNF; Trunc, Truncated; Veh, vehicle; ALA, alpha lipoic acid; BDNF, brain-derived neurotrophic factor; TrkB, tyrosine kinase B.

Next, we examined RR-EAE mice for microglial activation, an important player in MS-induced neuropathic pain (Khan and Smith 2014). For vehicle-treated RR-EAE mice at 35 d.p.i., microglial activation, as measured by CD11b or Iba-1 expression, in the lumbar spinal dorsal horn was approximately threefold higher (*F*_(2,33)_ = 45.57; *P *<* *0.05; Fig.3) *c.f*. vehicle-treated sham-mice, with a similar difference between the two groups when assessed by western blot (*F*_(2,6)_ = 29.82; *P *<* *0.05; Fig.4). However, for RR-EAE mice administered ALA at 10 mg kg^−1^ day^−1^ for 21 days, levels of microglial expression in the spinal dorsal horn did not differ significantly *c.f*. vehicle-treated sham-mice when assessed by IHC (*F*_(2,33)_ = 45.57; *P *>* *0.05) or western blot (*F*_(2,6)_ = 29.82; *P *>* *0.05).

#### BDNF and TrkB expression

BDNF-TrkB signaling in the spinal cord has been well established in peripheral neuropathic pain conditions (Vanelderen et al. 2010). Hence, we investigated expression levels of BDNF and TrkB in vehicle-treated RR-EAE mice exhibiting robust mechanical allodynia at 35 d.p.i. The mean IF levels for BDNF and TrkB in lumbar spinal dorsal horn of vehicle-treated RR-EAE mice were ∼2.5- (*F*_(2,33)_ = 15.84; *P *<* *0.05) and ∼3.2- (*F*_(2,33)_ = 71.52; *P *<* *0.05) fold higher *c.f*. their respective levels in vehicle-treated sham-mice. By contrast, for RR-EAE mice treated chronically with ALA at 10 mg kg^−1^ day^−1^, mean levels of BDNF and TrkB IF in lumbar spinal dorsal horn did not differ significantly (*P *>* *0.05) from the respective mean levels of BDNF and TrkB IF for vehicle-treated sham-mice (Fig.3).

Interestingly, using western blot, we found differential changes in lumbar spinal cord expression levels of the three BDNF isoforms, viz pro-BDNF (precursor of mature-BDNF), truncated-BDNF (intermediate protein), and mature-BDNF between treatment groups (Mowla et al. 2001). Specifically, for vehicle-treated RR-EAE mice, pro-BDNF and truncated-BDNF expression levels were increased significantly by ∼2.0- (*F*_(2,6)_ = 20.31; *P *<* *0.05) and ∼2.2- (*F*_(2,6)_ = 24.90; *P *<* *0.05) fold, respectively, *c.f*. the corresponding levels for vehicle-treated sham-mice. For RR-EAE mice, chronic treatment with ALA at 10 mg kg^−1^ day^−1^ normalized lumbar spinal cord expression levels of pro-BDNF and truncated-BDNF to match the corresponding levels for vehicle-treated sham-mice. By contrast, lumbar spinal cord expression levels of mature-BDNF did not differ significantly between the treatment groups (*F*_(2,6)_ = 1.79; *P* > 0.05; Fig.4).

Additionally, western blot analysis showed that expression levels of TrkB isoforms including truncated-TrkB and full-length TrkB were increased by ∼2.3- (*F*_(2,6)_ = 164.7; *P* < 0.05) and ∼4.0- (*F*_(2,6)_ = 125.2; *P* < 0.05) fold, respectively, in lumbar spinal cord of vehicle-treated RR-EAE mice *c.f*. the respective levels in vehicle-treated sham-mice. Chronic treatment of RR-EAE mice with ALA at 10 mg kg^−1^ day^−1^ normalized lumbar spinal cord expression levels of both TrkB isoforms to match the corresponding levels for vehicle-treated sham-mice (*P *>* *0.05; Fig.4).

#### Phospho-ERK (pERK)

Finally, we examined pERK in RR-EAE mice as a marker of neuronal and/or glial cell activation (Gao and Ji 2009; Yamamoto et al. 2013). For vehicle-treated RR-EAE mice exhibiting neuropathic pain behavior, mean pERK IF in sections of lumbar spinal dorsal horn (laminae I–II) was ∼3.6-fold higher (*F*_(2,33)_ = 42.49; *P *<* *0.05; Fig.3) *c.f*. the respective levels in vehicle-treated sham-mice, with a similar difference between the two groups when assessed by western blot (*F*_(2,6)_ = 16.43; *P *<* *0.05; Fig.4). By contrast, mean pERK expression levels in lumbar spinal dorsal horn of RR-EAE mice treated with ALA (10 mg kg^−1^ day^−1^) did not differ significantly from that for vehicle-treated sham-mice when assessed by IHC (*F*_(2,33)_ = 42.49; *P *>* *0.05; Fig.3) and western blot (*F*_(2,6)_ = 16.43; *F*; *P *>* *0.05; Fig.4). Also, there were no significant between-group differences (*F*_(2,6)_ = 4.06; *P *>* *0.05) in lumbar spinal cord expression levels of either total ERK or GAPDH (Fig.4).

### Immunohistology: colocalization of BDNF, TrkB, and pERK with CD3^+^ T-cells

Using specific IF antibodies, we show herein that BDNF, TrkB, and pERK were highly colocalized with CD3^+^ T cells in the dorsal horn of lumbar spinal cord sections from RR-EAE mice (Figs.8). These markers were also colocalized with subsets of neurons (NeuN) and microglia/macrophages (CD11b or Iba1) (Figs.8). BDNF and TrkB were minimally colocalized with astrocytes (GFAP), whereas pERK was colocalized with a subset of astrocytes (Figs.8). BDNF was highly colocalized with its receptor, TrkB, in the dorsal horn of lumbar spinal cord sections from these animals (Figs.8).

### Chronic ALA treatment: no signs of hepatic or renal toxicity

Mean (±SEM) plasma concentrations of various biochemical markers of hepatic and renal function (Table2) did not differ significantly (*F*_(2,9)_ = 0.11, ALT; *F*_(2,9)_ = 0.37, AST; *F*_(2,12)_ = 0.26, ALP; *F*_(2,9)_ = 2.43, total bilirubin; *F*_(2,12)_ = 1.68, Urea; (*P *>* *0.05) between RR-EAE mice administered ALA (10 mg kg^−1^ day^−1^) or vehicle for 21 consecutive days *c.f*. vehicle-treated sham-mice. Similarly, mean (±SEM) liver and kidney weights did not differ significantly *F*_(2,15)_ = 0.2, liver; *F*_(2,15)_ = 2.7, kidneys; *P *>* *0.05) between these three groups (Table2). At necropsy, there were no gross abnormalities observed in the brain, lungs, heart, spleen, liver, and kidneys, consistent with the absence of treatment-related adverse behavioral effects in either group of RR-EAE mice or sham-mice (Table2).

## Discussion

Here, we show novel findings that once-daily chronic treatment with ALA (3 or 10 mg kg^−1^ day^−1^) for 3 weeks attenuated the development of bilateral hindpaw hypersensitivity in an optimized RR-EAE mouse model of MS-induced neuropathic pain in a dose-dependent manner. Specifically, at 35 d.p.i., mean (±SEM) PWTs for RR-EAE mice administered chronic ALA at 10 mg kg^−1^ day^−1^ did not differ significantly (*P *>* *0.05) from the corresponding PWTs for vehicle-treated sham-mice. By contrast, for RR-EAE mice administered vehicle for 3 weeks, mechanical allodynia was fully developed in the bilateral hindpaws at 26 d.p.i. (*P *<* *0.05), that persisted until study completion (Fig.1).

Additionally, we showed that the efficacious chronic ALA dosing regimen at 10 mg kg^−1^ day^−1^ in RR-EAE mice was well tolerated. There were no gross abnormalities at necropsy and biochemical indices of hepatic and renal function were within the normal ranges (Table2). Interestingly, 3 weeks after cessation of the chronic ALA dosing regimen at 10 mg kg^−1^ day^−1^ in Cohort 2 RR-EAE mice, mechanical allodynia recurred in the bilateral hindpaws at 55 d.p.i. in a manner similar to that exhibited by vehicle-treated RR-EAE mice from 26 d.p.i. onward. These findings clearly suggest that ongoing chronic ALA treatment is required to prevent development of persistent MS-associated neuropathic pain (Fig.1).

Importantly, mechanical allodynia that developed in the bilateral hindpaws of these RR-EAE mice was unaffected by the relapsing-remitting clinical disease progression, in agreement with previous studies in this field (Aicher et al. 2004., Khan et al. 2014; Olechowski et al. 2009). Together, these findings suggest that distinct pathophysiological mechanisms underpin motor and sensory deficits in rodent models of EAE (Aicher et al. 2004; Khan et al. 2014).

Our investigations herein on the cellular and molecular mechanisms contributing to the progressive alleviation of hindpaw hypersensitivity in RR-EAE mice treated with ALA (10 mg kg^−1^ day^−1^), show that CD3^+^ T-cell infiltration into the lumbar spinal dorsal horn was markedly reduced relative to that for vehicle-treated RR-EAE mice exhibiting mechanical allodynia in their bilateral hindpaws (Fig.2). The augmented CD3^+^ T-cell infiltration into the lumbar spinal dorsal horn of vehicle-treated RR-EAE mice was accompanied by microglial activation, mirroring previous reports of activated microglia in the CNS of EAE mice exhibiting clinical disease (Murphy et al. 2010). Importantly, relief of hindpaw hypersensitivity in RR-EAE mice by the efficacious chronic ALA dosing regimen (10 mg kg^−1^ day^−1^) was accompanied by marked attenuation of microglial activation in the spinal dorsal horn (Figs.4). Our findings agree with previous work showing that ALA inhibits T-cell migration into the spinal cord of EAE mice (Morini et al. 2004; Wang et al. 2013).

Increased expression of BDNF and its high-affinity receptor, TrkB, has been widely implicated in the pathobiology of peripheral neuropathic pain conditions leading to the development of central sensitization in the dorsal horn of the spinal cord (Geng et al. 2010). Indeed, this is regarded as a pathobiologic hallmark of peripheral neuropathic pain, characterized by increased responsiveness of nociceptive neurons in the CNS to normal or subthreshold afferent input (Merskey and Bogduk 1994). However, the possibility that upregulated BDNF-TrkB-ERK signaling in the spinal dorsal horn may contribute to the pathobiology of MS-associated CNP, has not hitherto been investigated.

**Figure 5:**
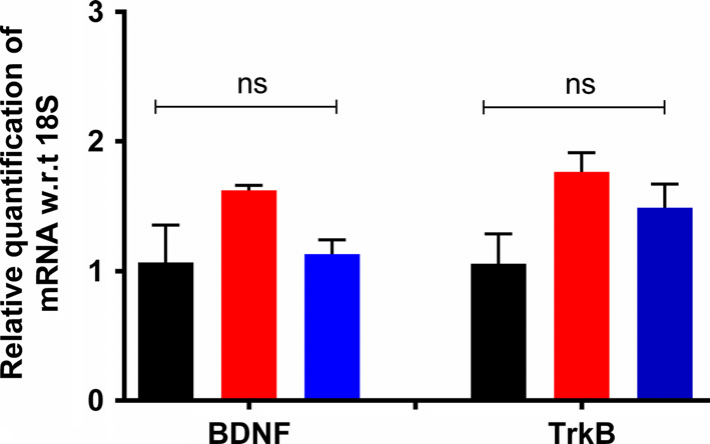
Expression levels of mRNA for BDNF and TrkB in lumbar (L4-L6) spinal cord of RR-EAE mice administered either ALA at 10 mg kg^−1^ day^−1^ or vehicle for 3 weeks did not differ significantly (*F*_(2,8)_ = 3.8; BDNF and *F*_(2,7)_ = 3.59; *P *>* *0.05) from the respective data for the lumbar spinal cord of vehicle-treated sham-mice (one-way ANOVA followed by Tukey’s multiple comparison test). EAE, experimental autoimmune encephalomyelitis, Veh, vehicle; ALA, alpha lipoic acid; BDNF, brain-derived neurotrophic factor; TrkB, tyrosine kinase B; ANOVA, analysis of variance.

Herein, we showed that for vehicle-treated RR-EAE mice with fully developed mechanical allodynia in the bilateral hindpaws, lumbar spinal dorsal horn expression levels of BDNF and TrkB were markedly increased compared with the corresponding expression levels in vehicle-treated sham-mice (Figs.4). However, lumbar spinal cord expression levels of mRNA for BDNF and TrkB in our RR-EAE-mouse model of MS-neuropathic pain did not differ significantly (*P *>* *0.05) from the respective levels for vehicle-treated sham-mice (Fig.5). Our findings are aligned with reports by others of unchanged mRNA levels for BDNF and TrkB in the CNS of EAE mice exhibiting clinical disease (Zhu et al. 2012, 2014).

Elevated expression levels of BDNF and TrkB in the lumbar spinal cord of RR-EAE mice may be due to increased translation of BDNF and TrkB from mRNA in spinal cord neurons and/or glia. Alternatively, elevated spinal cord expression levels of BDNF and TrkB may be via a mechanism not involving enhanced local synthesis (Tonra et al. 1998). This latter notion is supported by reports of upregulated BDNF expression by infiltrating T-cells due to antigen stimulation and/or secondary to demyelination of CNS neurons (Stadelmann et al. 2002; Gielen et al. 2003). Although infiltrating T-cell-derived BDNF was originally proposed to be neuroprotective in the CNS of EAE mice (Moalem et al. 2000; Stadelmann et al. 2002), more recent work suggests that T-cell-derived BDNF is not critical for neuroprotective effects in CNS of EAE mice (Lee et al. 2012; Xin et al. 2012).

Our present findings of differential changes in expression levels of BDNF isoforms in the lumbar spinal cord of vehicle-treated RR-EAE mice *c.f*. vehicle-treated sham-mice, are novel (Fig.4). Specifically, there was significant upregulation of pro-BDNF and truncated-BDNF expression in lumbar spinal cord of vehicle-treated RR-EAE mice exhibiting neuropathic pain behavior. However, although there was a trend for reduced mature-BDNF expression levels in vehicle-treated RR-EAE mice, it was not statistically significant (*P *>* *0.05) relative to the respective levels for vehicle-treated sham-mice. Notably, the dysregulated expression of BDNF as well as TrkB isoforms was normalized in RR-EAE mice administered chronic ALA treatment at 10 mg kg^−1^ day^−1^ for 3-weeks (Fig.4).

Our finding of upregulated total BDNF (Figs.3, S1) in the lumbar spinal dorsal horn of vehicle-treated RR-EAE mice is consistent with previous reports of upregulated levels of BDNF in EAE mice as well as CSF and/or plasma levels from patients with RR-MS (Sarchielli et al. 2002; Frota et al. 2009). Our novel findings herein of differential changes in BDNF isoforms in the lumbar spinal cord of RR-EAE mice are aligned with a recent report of dysregulated serum levels of BDNF isoforms in patients with RR-MS (Tongiorgi et al. 2012). Hence, future investigation on the extent to which differential changes in serum levels of the various BDNF isoforms is a marker for MS-associated neuropathic pain in humans, is warranted.

A biological role for truncated-BDNF is currently unclear, whereas pro- and mature-BDNF are considered ‘Yin and Yang’ molecules due to their opposing biological roles (Chao and Bothwell 2002). Mature-BDNF signaling via TrkB has a role in cell survival and long-term potentiation in the CNS, whereas pro-BDNF is associated with apoptosis and long-term depression mediated by p75^NTR^ activation in the CNS (Chao and Bothwell 2002; Stadelmann et al. 2002). A pronociceptive role for pro-BDNF signaling via its main receptor, p75^NTR^, is suggested by the presence of both pro-BDNF and p75^NTR^ in the superficial layers of the spinal dorsal horn together with its anterograde transport to nerve terminals (King et al. 2000; Zhou et al. 2004) and co-release with mature-BDNF from activated microglia (Srinivasan et al. 2004).

**Figure 6:**
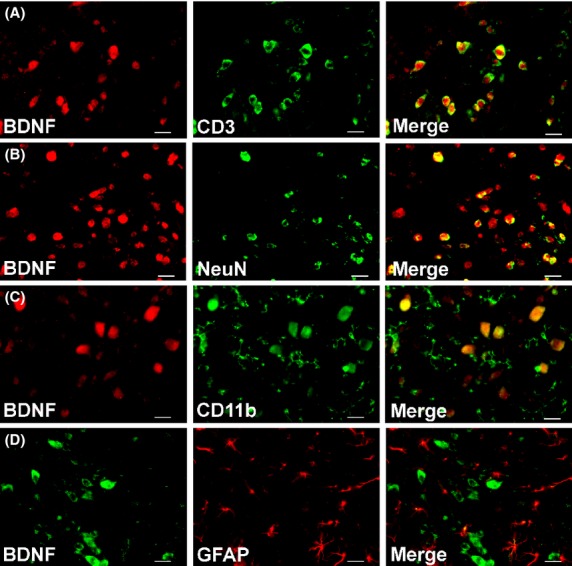
In the dorsal horn of lumbar (L4-L6) spinal cord of vehicle-treated RR-EAE mice, BDNF is colocalized predominantly with (A) CD3^+^ T cells. It is also colocalized with a subset of (B) neurons (NeuN) and to a lesser extent with (C) microglia/macrophages (CD11b) and (D) minimally with astrocytes (GFAP). Scale bars represent 20 *μ*m.

Our data showing concurrent increases in pERK and pro-BDNF expression levels in vehicle-treated RR-EAE mice (Figs.4) are aligned with previous work by others showing that concentration-dependent activation of TrkB by pro-BDNF, albeit with lower affinity than mature-BDNF, elicits prototypical TrkB downstream signaling including ERK activation (Fayard et al. 2005; Boutilier et al. 2008; Sakuragi et al. 2013). As pERK is regarded as a marker of central sensitization in peripheral neuropathic pain conditions (Zhuang et al. 2005; Gao and Ji 2009), it may also have a pathobiologic role in CNP conditions such as MS-associated neuropathic pain. In vehicle-treated RR-EAE mice herein, upregulated expression levels of BDNF, TrkB, and pERK were colocalized primarily with infiltrated CD3^+^ T cells in the lumbar spinal dorsal horn. These markers were also colocalized with subsets of neurons and to a lesser extent with microglia/macrophages. However, there was minimal colocalization with astrocytes for BDNF and TrkB (Figs.6, 8). Hence, it is plausible that augmented levels of pro-BDNF signaling, at least in part, via full-length TrkB to increase pERK expression levels in the spinal dorsal horn of vehicle-treated RR-EAE mice, may contribute to the development of hindpaw hypersensitivity in these mice.

**Figure 7:**
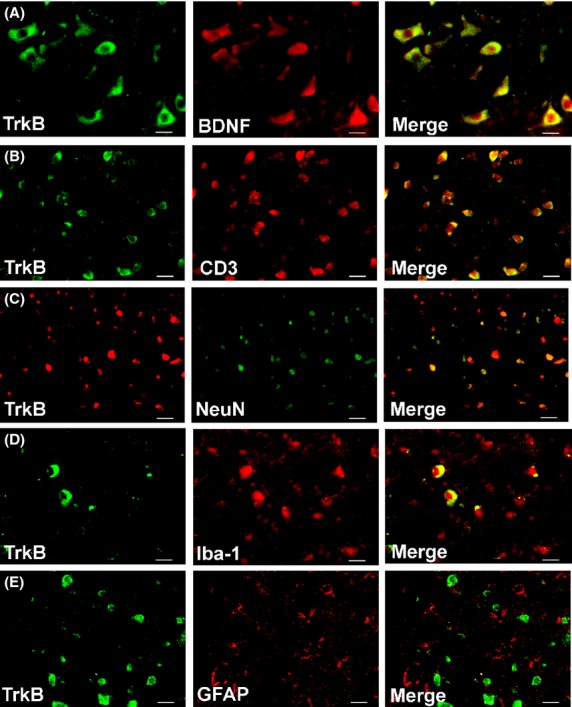
In the dorsal horn of lumbar (L4-L6) spinal cord of vehicle-treated RR-EAE mice, TrkB is colocalized predominantly with (A) BDNF and (B) CD3^+^ T cells. It is also colocalized with a subset of (C) neurons (NeuN) and to a lesser extent with (D) microglia/macrophages (Iba-1) and (E) minimally with astrocytes (GFAP). Scale bars represent 20 *μ*m; BDNF, brain-derived neurotrophic factor; TrkB, tyrosine kinase B.

**Figure 8:**
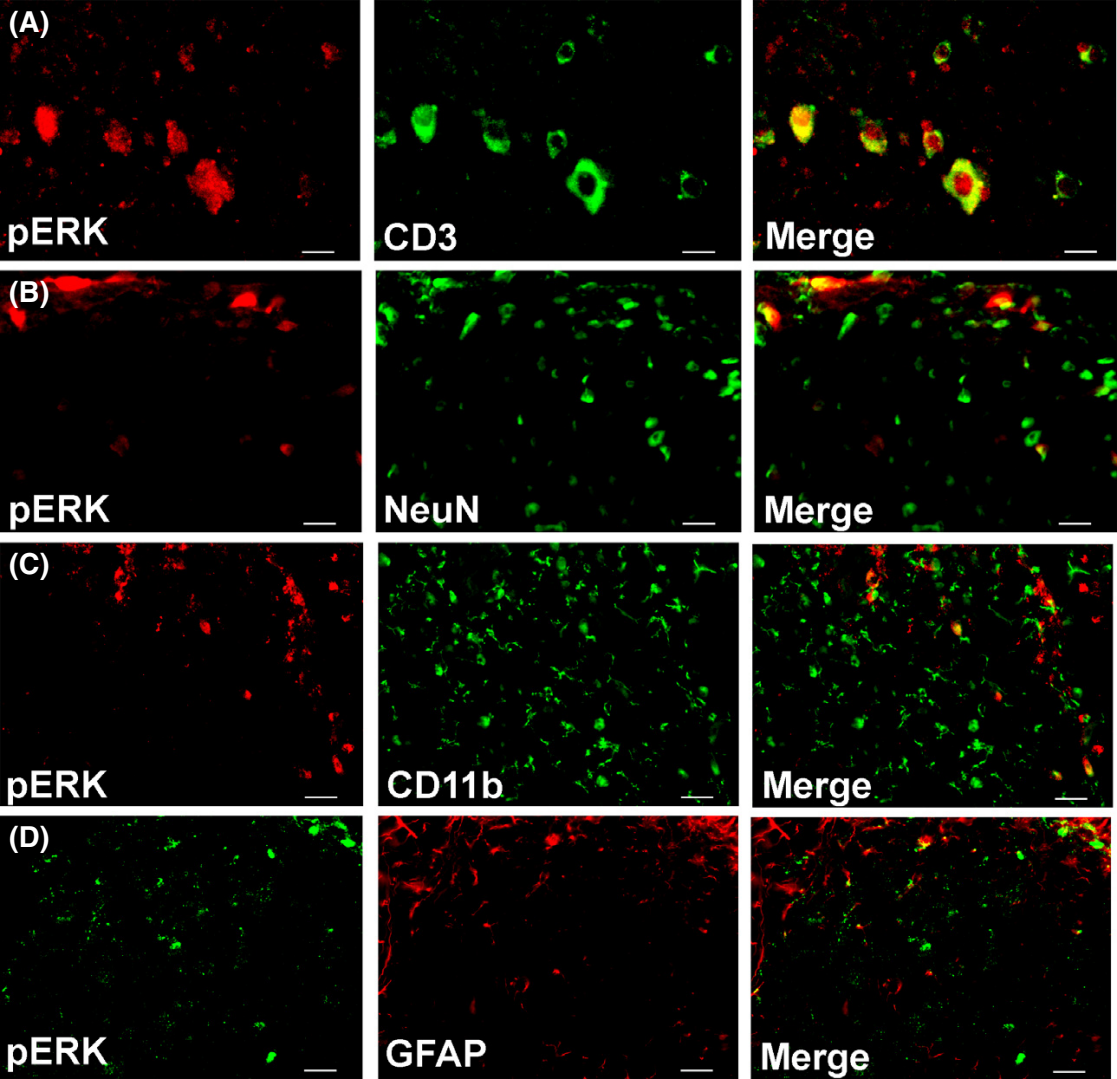
In the dorsal horn of lumbar (L4-L6) spinal cord of vehicle-treated RR-EAE mice, pERK is colocalized predominantly with (A) CD3^+^ T cells. It is also colocalized with a subset of (B) neurons (NeuN) and to a lesser extent with (C) microglia/macrophages (CD11b) and (D) a subset of astrocytes (GFAP). Scale bars represent 20 *μ*m.

## Conclusions

Our findings demonstrate inhibition of the development of mechanical allodynia in the bilateral hindpaws of RR-EAE mice by once-daily treatment with ALA at 10 mg kg^−1^ day^−1^ for 3 weeks. The cellular and molecular mechanisms underpinning the antiallodynic effects of ALA appear to involve a marked reduction in upregulated expression levels of BDNF-, TrkB-, and pERK into the dorsal horn of the lumbar spinal cord of RR-EAE mice, to match the respective levels for vehicle-treated sham-mice.

## Abbreviations

ALA: alpha lipoic acid
BDNF: brain-derived neurotrophic factor
BLOQ: below lower limit of quantification.
CIPN: chemotherapy-induced peripheral neuropathy
CNP: central neuropathic pain
CNS: central nervous system
DAPI: 4′,6-diamidino-2-phenylindole dihydrochloride
IF: immunofluoresence
IHC: immunohistochemistry
MAPK: mitogen-activated protein kinase
MS: multiple sclerosis
PBS: phosphate-buffered saline
PWT: paw withdrawal threshold
RR-EAE: relapsing-remitting experimental autoimmune encephalomyelitis
TrkB: tyrosine kinase B

## Supporting Information

Supplementary methods.

**Figure S1.** Total BDNF (pro-, truncated-, and mature-BDNF) expression by western blot in lumbar (L4-L6) spinal cord of RR-EAE mice administered ALA at 10 mg kg^−1^ day^−1^ or vehicle, for 3 weeks relative to the respective data for the lumbar spinal cord of vehicle-treated sham-mice. Specifically, for vehicle-treated RR-EAE mice there was ∼1.8-fold (*F*_(2,6)_ = 7.27; *P *<* *0.05) increase in total BDNF *c.f*. the respective data for lumbar spinal cord of vehicle-treated sham-mice. For RR-EAE mice treated with ALA (10 mg kg^−1^ day^−1^) for 21 days, there were no significant differences (*P *>* *0.05) in expression levels of total BDNF from the respective data for the lumbar spinal cord of vehicle-treated sham-mice (one-way ANOVA followed by Tukey’s multiple comparison test). EAE, experimental autoimmune encephalomyelitis, Veh, vehicle.

**Figure S2.** Immunohistochemical (IHC) analysis of CD11b, BDNF, TrkB, and pERK in the dorsal horn of lumbar (L4-L6) spinal cord sections from RR-EAE mice administered ALA at 10 mg kg^−1^ day^−1^ or vehicle, for 3 weeks relative to the corresponding data for vehicle-treated sham-mice. (A) A representative image of lumbar spinal cord showing the region (yellow dotted lines) of the dorsal horn targeted uniformly during all IHC analyses. (B) The extent of microglia/macrophage (CD11b) activation (approximately threefold; *F*_(2,33)_ = 45.57; *P *<* *0.05), as well as expression levels of (C) BDNF (∼2.5-fold; *F*_(2,33)_ = 15.84; *P *<* *0.05); (D) TrkB (∼3.2-fold; *F*_(2,33)_ = 71.52; *P *<* *0.05) and (E) pERK (∼3.6-fold; *F*_(2,33)_ = 42.49; *P *<* *0.05) was significantly increased in vehicle-treated RR-EAE mice *c.f*. the respective data for the lumbar spinal cord of vehicle-treated sham-mice. For RR-EAE mice treated with ALA (10 mg kg^−1^ day^−1^), lumbar spinal cord expression levels of CD11b, BDNF, TrkB, and pERK did not differ significantly (*P *>* *0.05) from the respective data for the lumbar spinal cord of vehicle-treated sham-mice (one-way ANOVA followed by Tukey’s multiple comparison test). Scale bars: 200 *μ*m for (A) and 100 *μ*m for (B–E). EAE, experimental autoimmune encephalomyelitis, Veh, vehicle; ALA, alpha lipoic acid.

## References

[b501] Aicher SA, Silverman MB, Winkler CW, Bebo BF (2004). Hyperalgesia in an animal model of multiple sclerosis. Pain.

[b1] Alexander SP, Benson HE, Faccenda E, Pawson AJ, Sharman JL, McGrath JC (2013). The Concise Guide to PHARMACOLOGY 2013/14: overview. Br J Pharmacol.

[b2] Amor S, Baker D (2012). Checklist for reporting and reviewing studies of experimental animal models of multiple sclerosis and related disorders. Mult Scler Relat Disord.

[b3] Boutilier J, Ceni C, Pagdala PC, Forgie A, Neet KE, Barker PA (2008). Proneurotrophins require endocytosis and intracellular proteolysis to induce TrkA activation. J Biol Chem.

[b4] Chao MV, Bothwell M (2002). Neurotrophins: to cleave or not to cleave. Neuron.

[b5] Chaplan SR, Bach FW, Pogrel JW, Chung JM, Yaksh TL (1994). Quantitative assessment of tactile allodynia in the rat paw. J Neurosci Methods.

[b6] Compston A, Coles A (2008). Multiple sclerosis. Lancet.

[b7] Dworkin RH, Levy RM, Mackey SC, Mayer J, Miaskowski C, Raja SN (2010). Recommendations for the pharmacological management of neuropathic pain: an overview and literature update. Mayo Clin Proc.

[b8] Fayard B, Loeffler S, Weis J, Vögelin E, Krüttgen A (2005). The secreted brain-derived neurotrophic factor precursor pro-BDNF binds to TrkB and p p75^NTR^ but not to TrkA or TrkC. J Neurosci Res.

[b9] Fleming KK, Bovaird JA, Mosier MC, Emerson MR, LeVine SM, Marquis JG (2005). Statistical analysis of data from studies on experimental autoimmune encephalomyelitis. J Neuroimmunol.

[b10] Frota ERC, Rodrigues DH, Donadi EA, Brum DG, Maciel DRK, Teixeira AL (2009). Increased plasma levels of brain derived neurotrophic factor (BDNF) after multiple sclerosis relapse. Neurosci Lett.

[b11] Gao YJ, Ji RR (2009). c-Fos and pERK, which is a better marker for neuronal activation and central sensitization after noxious stimulation and tissue injury?. Open Pain J.

[b12] Geng S-J, Liao F-F, Dang W-H, Ding X, Liu X-D, Cai J (2010). Contribution of the spinal cord BDNF to the development of neuropathic pain by activation of the NR2B-containing NMDA receptors in rats with spinal nerve ligation. Exp Neurol.

[b13] Gielen A, Khademi M, Muhallab S, Olsson T, Piehl F (2003). Increased brain-derived neurotrophic factor expression in white blood cells of relapsing-remitting multiple sclerosis patients. Scand J Immunol.

[b14] Jain MR, Li Q, Liu T, Rinaggio J, Ketkar A, Tournier V (2012). Proteomic identification of immunoproteasome accumulation in formalin-fixed rodent spinal cords with experimental autoimmune encephalomyelitis. J Proteome Res.

[b15] Kalia LV, O’Connor PW (2005). Severity of chronic pain and its relationship to quality of life in multiple sclerosis. Mult scler.

[b16] Khan N, Smith MT (2014). Multiple sclerosis-induced neuropathic pain: pharmacological management and pathophysiological insights from rodent EAE models. Inflammopharmacology.

[b17] Khan N, Woodruff TM, Smith MT (2014). Establishment and characterization of an optimized mouse model of multiple sclerosis-induced neuropathic pain using behavioral, pharmacologic, histologic and immunohistochemical methods. Pharmacol Biochem Behav.

[b18] Kilkenny C, Browne W, Cuthill IC, Emerson M, Altman DG, Altman DG, Group NCRRGW (2010). Animal research: reporting in vivo experiments: the ARRIVE guidelines. Br J Pharmacol.

[b19] King VR, Bradbury EJ, McMahon SB, Priestley JV (2000). Changes in truncated trkB and p75 receptor expression in the rat spinal cord following spinal cord hemisection and spinal cord hemisection plus neurotrophin treatment. Exp Neurol.

[b20] Lee DH, Geyer E, Flach AC, Jung K, Gold R, Flugel A (2012). Central nervous system rather than immune cell-derived BDNF mediates axonal protective effects early in autoimmune demyelination. Acta Neuropathol.

[b21] Lee JS, Kim YH, Kim DB, Bang WS, Lee OH (2014). Acute and 4-week repeated-dose oral toxicity studies of Cirsium setidens in rats. Molecules.

[b22] Merskey H, Bogduk N (1994). Classification of chronic pain. Descriptors of chronic pain syndromes and definitions of pain terms.

[b23] Moalem G, Gdalyahu A, Shani Y, Otten U, Lazarovici P, Cohen IR (2000). Production of neurotrophins by activated T cells: implications for neuroprotective autoimmunity. J Autoimmun.

[b24] Morgado C, Pereira-Terra P, Tavares I (2011). Alpha-Lipoic acid normalizes nociceptive neuronal activity at the spinal cord of diabetic rats. Diabetes Obes Metab.

[b25] Morini M, Roccatagliata L, Dell’Eva R, Pedemonte E, Furlan R, Minghelli S (2004). Alpha-lipoic acid is effective in prevention and treatment of experimental autoimmune encephalomyelitis. J Neuroimmunol.

[b26] Mowla SJ, Farhadi HF, Pareek S, Atwal JK, Morris SJ, Seidah NG (2001). Biosynthesis and post-translational processing of the precursor to brain-derived neurotrophic factor. J Biol Chem.

[b502] Murphy AC, Lalor SJ, Lynch MA, Mills KH (2010). Infiltration of Th1 and Th17 cells and activation of microglia in the CNS during the course of experimental autoimmune encephalomyelitis. Brain Behav Immun.

[b27] NHMRC (National Health and Medical Research Council) (2004). Australian code of practice for the care and use of animals for scientific purposes.

[b28] NHMRC (National Health and Medical Research Council) (2013). Australian code of practice for the care and use of animals for scientific purposes.

[b503] Olechowski CJ, Truong JJ, Kerr BJ (2009). Neuropathic pain behaviours in a chronic-relapsing model of experimental autoimmune encephalomyelitis (EAE). Pain.

[b29] Osterberg A, Boivie J, Thuomas KA (2005). Central pain in multiple sclerosis-prevalence and clinical characteristics. Eur J Pain.

[b504] Peiris M, Monteith GR, Roberts-Thomson SJ, Cabot PJ (2007). A model of experimental autoimmune encephalomyelitis (EAE) in C57BL/6 mice for the characterisation of intervention therapies. J Neurosci Methods.

[b30] Sakuragi S, Tominaga-Yoshino K, Ogura A (2013). Involvement of TrkB- and p75NTR-signaling pathways in two contrasting forms of long-lasting synaptic plasticity. Sci Rep.

[b31] Sarchielli P, Greco L, Stipa A, Floridi A, Gallai V (2002). Brain-derived neurotrophic factor in patients with multiple sclerosis. J Neuroimmunol.

[b32] Schmittgen TD, Livak KJ (2008). Analyzing real-time PCR data by the comparative CT method. Nat Protoc.

[b33] Srinivasan B, Roque CH, Hempstead BL, Al-Ubaidi MR, Roque RS (2004). Microglia-derived pronerve growth factor promotes photoreceptor cell death via p75 neurotrophin receptor. J Biol Chem.

[b34] Stadelmann C, Kerschensteiner M, Misgeld T, Bruck W, Hohlfeld R, Lassmann H (2002). BDNF and gp145trkB in multiple sclerosis brain lesions: neuroprotective interactions between immune and neuronal cells?. Brain.

[b35] Tongiorgi E, Sartori A, Baj G, Bratina A, Di Cola F, Zorzon M (2012). Altered serum content of brain-derived neurotrophic factor isoforms in multiple sclerosis. J Neurol Sci.

[b36] Tonra JR, Curtis R, Wong V, Cliffer KD, Park JS, Timmes A (1998). Axotomy upregulates the anterograde transport and expression of brain-derived neurotrophic factor by sensory neurons. J Neurosci.

[b37] Trevisan G, Materazzi S, Fusi C, Altomare A, Aldini G, Lodovici M (2013). Novel therapeutic strategy to prevent chemotherapy-induced persistent sensory neuropathy by TRPA1 blockade. Cancer Res.

[b38] Vanelderen P, Rouwette T, Kozicz T, Roubos E, Van Zundert J, Heylen R (2010). The role of brain-derived neurotrophic factor in different animal models of neuropathic pain. Eur J Pain.

[b39] Wang KC, Tsai CP, Lee CL, Chen SY, Lin GJ, Yen MH (2013). *α*-Lipoic acid enhances endogenous peroxisome-proliferator-activated receptor-gamma to ameliorate experimental autoimmune encephalomyelitis in mice. Clin Sci (Lond).

[b505] Watson JL, Hala TJ, Putatunda R, Sannie D, Lepore AC (2014). Persistent at-level thermal hyperalgesia and tactile allodynia accompany chronic neuronal and astrocyte activation in superficial dorsal horn following mouse cervical contusion spinal cord injury. PLoS ONE.

[b40] Wu J, Ohlsson M, Warner EA, Loo KK, Hoang TX, Voskuhl RR (2008). Glial reactions and degeneration of myelinated processes in spinal cord gray matter in chronic experimental autoimmune encephalomyelitis. Neuroscience.

[b41] Xin J, Mesnard NA, Beahrs T, Wainwright DA, Serpe CJ, Alexander TD (2012). CD4 +  T cell-mediated neuroprotection is independent of T cell-derived BDNF in a mouse facial nerve axotomy model. Brain Behav Immun.

[b43] Yamamoto S, Kishishita Y, Yoshida M, Miura D, Suzuki H, Ishikawa K (2013). Activation of different signals identified with glia cells contribute to the progression of hyperalgesia. Cell Mol Neurobiol.

[b506] Zhou X, Hansson GK (2004). Effect of sex and age on serum biochemical reference ranges in C57BL/6J mice. Comp Med.

[b44] Zhou XF, Song XY, Zhong JH, Barati S, Zhou FH, Johnson SM (2004). Distribution and localization of pro-brain-derived neurotrophic factor-like immunoreactivity in the peripheral and central nervous system of the adult rat. J Neurochem.

[b45] Zhu W, Frost EE, Begum F, Vora P, Au K, Gong Y (2012). The role of dorsal root ganglia activation and brain-derived neurotrophic factor in multiple sclerosis. J Cell Mol Med.

[b46] Zhu W, Acosta C, MacNeil BJ, Klonisch T, Cortes C, Doupe M (2014). Spinal cord brain derived neurotrophic factor (BDNF) responsive cells in an experimental autoimmune encephalomyelitis (EAE) model of multiple sclerosis (MS): implications in myelin repair. Res Immunol Int J.

[b47] Zhuang ZY, Gerner P, Woolf CJ, Ji RR (2005). ERK is sequentially activated in neurons, microglia, and astrocytes by spinal nerve ligation and contributes to mechanical allodynia in this neuropathic pain model. Pain.

