# Supplementary material for: Antiallodynic effects of alpha lipoic acid in an optimized RR-EAE mouse model of MS-neuropathic pain are accompanied by attenuation of upregulated BDNF-TrkB-ERK signaling in the dorsal horn of the spinal cord
Source: Pharmacol Res Perspect. 2015 May 4;3(3):e00137. doi: 10.1002/prp2.137 (PMC4492753; doi:10.1002/prp2.137)
Supplement: Supplementary file 1 [file prp20003-e00137-sd1.docx]

**Supplementary Materials and Methods for our Manuscript Entitled:**

**Antiallodynic effects of alpha lipoic acid in an optimized RR-EAE mouse model of MS-neuropathic pain are accompanied by attenuation of upregulated BDNF-TrkB-ERK signaling in the dorsal horn of the spinal cord**

Nemat Khan^1, 2^, Richard Gordon^3^, Trent M. Woodruff ^3^ and Maree T. Smith^1, 2^*

^1^The University of Queensland, Center for Integrated Preclinical Drug Development, St Lucia Campus, Brisbane, Queensland 4072, Australia.

^2^School of Pharmacy, The University of Queensland, Pharmacy Australia Center of Excellence, Woolloongabba, Brisbane, Queensland 4102, Australia.

^3^The School of Biomedical Sciences, University of Queensland, St Lucia Campus, Brisbane, Queensland 4072, Australia.

*Corresponding author

**Correspondence:**

Professor Maree T. Smith,

The University of Queensland,

Center for Integrated Preclinical Drug Development,

Level 3, Steele Building,

St Lucia Campus, Brisbane, Queensland 4072,

Australia.

Phone: +61 7-336-52554

Fax: +61-7-33467391

*E-mail:* [*maree.smith@uq.edu.au*](mailto:maree.smith@uq.edu.au)

**Mouse plasma biochemistry and organ weights**

At 35 d.p.i., blood samples were collected by cardiac puncture from euthanized RR-EAE or sham-mice in each treatment group (n=4-5/group), and immediately centrifuged at 3000 × g for 15 min. The separated plasma (~0.5 mL) samples were transferred to clean pre-labelled tubes and stored at -20^0^C. Subsequently, plasma samples were thawed and analysed using a Roche-Cobas Integra 800 auto-analyser (Roche Diagnostics GmbH, Mannheim, Germany) for concentrations of alanine transaminase (ALT), aspartate transaminase (AST), alkaline phosphatase (ALP), total bilirubin, urea and creatinine (Lee et al. 2014). After blood sample collection, these euthanized mice (n=6/group) underwent gross necropsy examination of the brain, liver, kidney, lungs, heart, and spleen. The liver and kidneys were also weighed individually.
