# Supplementary figures and images for: Antiallodynic effects of alpha lipoic acid in an optimized RR-EAE mouse model of MS-neuropathic pain are accompanied by attenuation of upregulated BDNF-TrkB-ERK signaling in the dorsal horn of the spinal cord
Source: Pharmacol Res Perspect. 2015 May 4;3(3):e00137. doi: 10.1002/prp2.137 (PMC4492753; doi:10.1002/prp2.137)

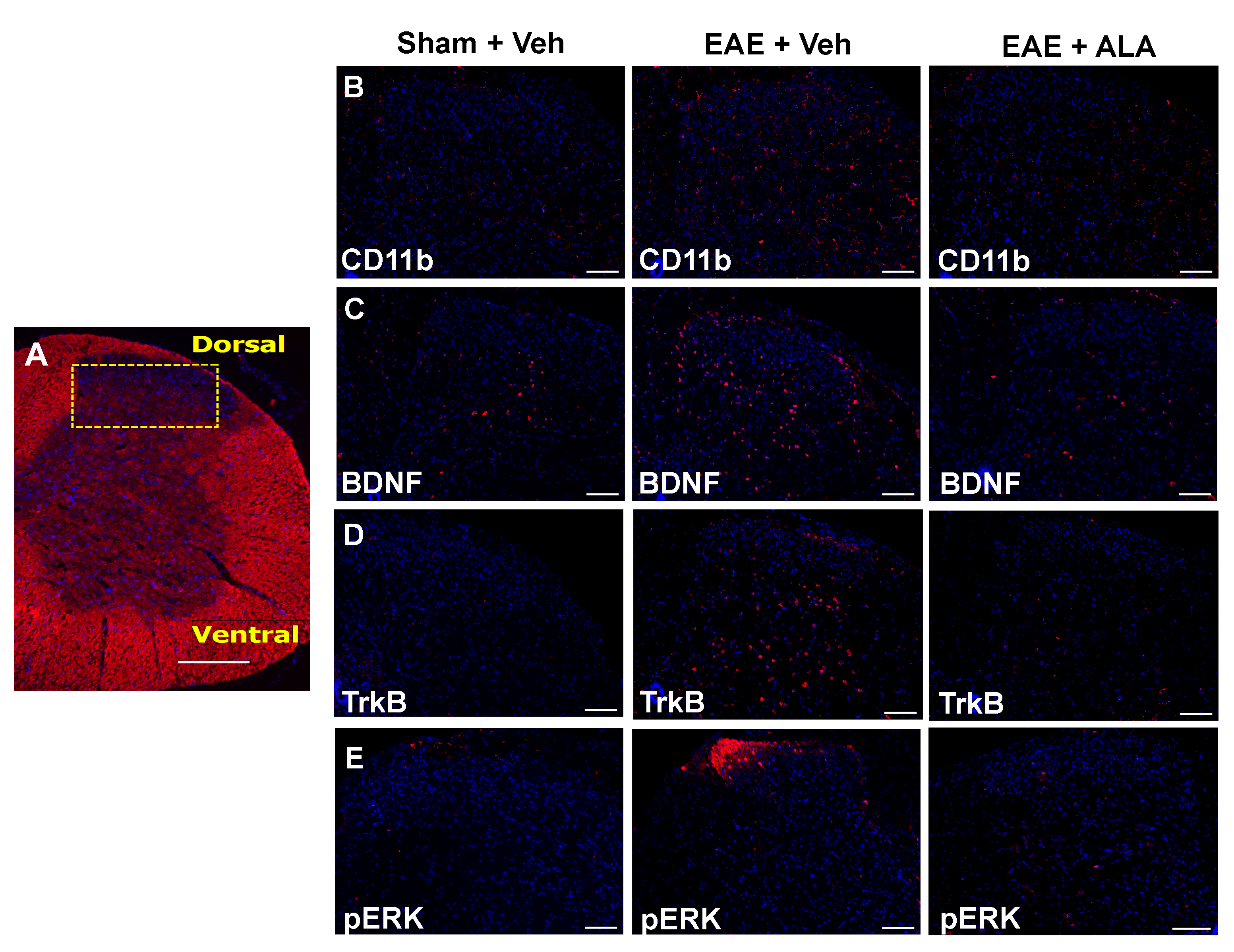

Supplement: Supplementary file 3 [file prp20003-e00137-sd3.tif]
